# Advances in GelMA Hydrogel-Enabled Angiogenic–Osteogenic Coupling: From Structural Programming to Exogenous Cue Synergy

**DOI:** 10.3390/jfb17060281

**Published:** 2026-06-06

**Authors:** Chenyujun Hu, Meng Zhang, Haoran Jiang, Yang Qu, Qi Meng, Jinqiu Tian, Hanran Zhang, Zhixiang Yang, Zhihao Lin, Bohan Xing, Peixun Zhang

**Affiliations:** 1 Department of Orthopedics and Trauma, Peking University People’s Hospital, Beijing 100044, China; 2511110405@bjmu.edu.cn (C.H.); 2211110413@stu.pku.edu.cn (H.J.); 2311110437@stu.pku.edu.cn (Y.Q.); 2511110399@bjmu.edu.cn (Q.M.); 20259023022@stumail.hbu.edu.cn (J.T.); 25032100305@stu.hebmu.edu.cn (H.Z.); 2023010566@ybu.edu.cn (Z.Y.); 2511210289@stu.pku.edu.cn (Z.L.); 2411210321@bjmu.edu.cn (B.X.); 2 National Centre for Trauma Medicine, Beijing 100044, China; 3 Key Laboratory of Trauma and Neural Regeneration, Peking University, Beijing 100044, China; 4 Beijing Laboratory of Trauma and Nerve Regeneration, Peking University, Beijing 100044, China; 5 Department of Trauma & Orthopedics, Peking University People’s Hospital Qingdao Hospital, Qingdao 266111, China

**Keywords:** GelMA hydrogel, angiogenic–osteogenic coupling, vascularized bone regeneration, functional biomaterials, tissue engineering, biomaterial design, stimuli-responsive hydrogels, bone regeneration

## Abstract

Vascular–osteogenic coupling plays a central regulatory role in bone regeneration, but it is frequently impaired under pathological conditions, including aging, ischemia, and chronic inflammation, which compromises efficient bone repair. Gelatin methacryloyl (GelMA) hydrogels, which combine extracellular matrix-like bioactivity, adjustable mechanical properties, and compatibility with three-dimensional biomanufacturing, have become a widely used material platform for vascularized bone regeneration. From the perspective of vascular–osteogenic coupling, this review reframes and synthesizes GelMA-based approaches for vascularized bone regeneration, grouping existing strategies into three categories: (i) intrinsic material design, in which pore architecture, microchannels, dynamic networks, and interfacial functionalization are used to guide vascular ingrowth; (ii) exogenous bioactive delivery, involving growth factors, extracellular vesicles, cells, and inorganic ions to enhance vascularization; and (iii) smart responsive strategies, including ROS/pH-responsive systems, sequential release, and external stimulation, which aim to recapitulate the evolving microenvironment during bone repair. This review further compares these strategies in terms of evidence level, reproducibility, and translational potential. Exogenous delivery systems currently have the strongest preclinical support, but issues related to dose standardization, burst release, and long-term safety remain unresolved. Intrinsic material programming is less extensively studied, yet may be more compatible with manufacturing consistency, sterilization, and engineering translation. Most stimuli-responsive systems, by contrast, remain largely at the small-animal or proof-of-concept stage. Future GelMA-based systems should therefore shift from increasing functional complexity toward improving predictability, reproducibility, and clinical feasibility. Compositionally defined and structurally controllable GelMA composites that integrate vascular regulation with mechanical support may provide a more realistic path for vascularized bone regeneration.

## 1. Introduction

Stable bone repair cannot be achieved through osteogenic induction alone, as this process also depends on vascular support. In bone tissue, blood vessels not only deliver oxygen and nutrients, but also regulate osteoprogenitor recruitment, differentiation, and functional status through vascular subtypes, flow-induced shear stress, and endothelial-cell-derived signals. Osteogenic cell lineages further contribute to the coordinated regulation of bone homeostasis and repair [1,2,3]. Bone vasculature is highly heterogeneous, and type H vessels are closely associated with osteoprogenitor cells and osteoblasts, playing a key role in maintaining osteogenic activity [4,5]. At the same time, osteoprogenitor cells can, in turn, regulate vascular formation, coordinating vascular development with bone formation [6,7,8]. At the molecular level, this coupling is regulated by the HIF-1α/VEGF axis, flow-mediated Dll4–Notch signaling, and endothelial–osteogenic paracrine interactions. HIF-1αstabilization promotes angiogenesis and bone formation, whereas blood-flow changes influence vascular function and osteogenesis through Notch signaling [9,10,11]. In addition, blocking VEGFR1/VEGFR2 expressed by osteoblasts can inhibit both angiogenesis and bone formation [12,13,14,15]. Pathological factors such as aging and chronic inflammation impair osteoprogenitor function, reduce type H vessels, and decrease perfusion. In parallel, abnormal endothelial signaling and immune imbalance further disturb the coordination between angiogenesis and bone formation [16,17,18].The key biological cues, regulatory axes, and corresponding design implications involved in angiogenic–osteogenic coupling are summarized in Table 1 and with more detailed comparative information provided in Supplementary Table S1.

**Figure 1:**
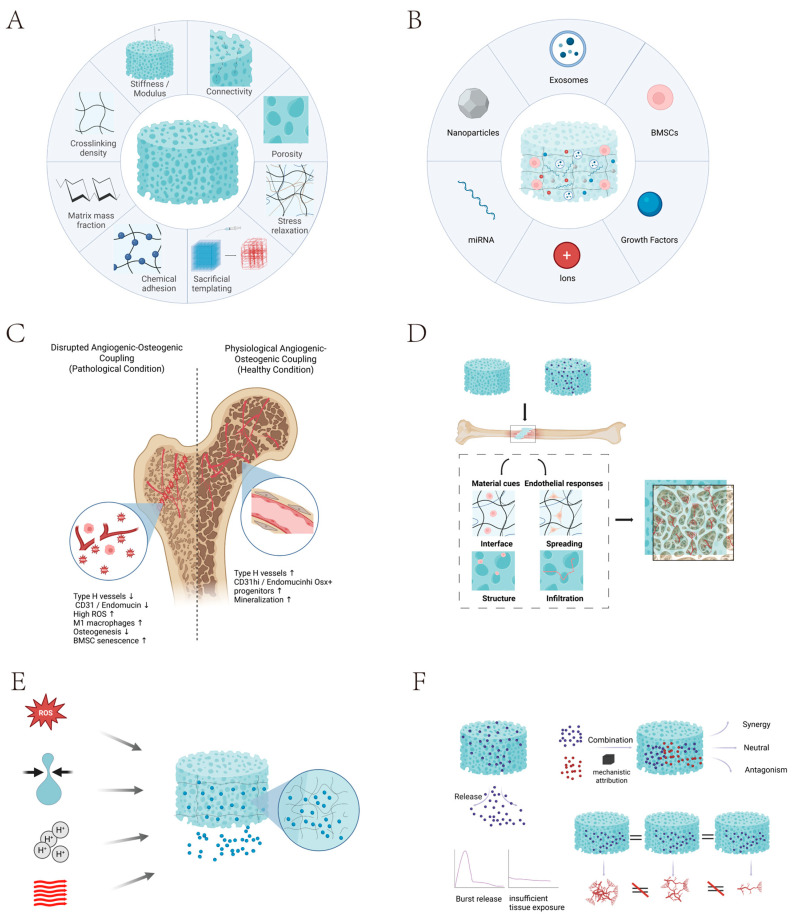
Schematic illustration of material strategies and an exogenous-synergy framework using GelMA hydrogels to re-establish angiogenic–osteogenic coupling. (**A**). Intrinsic physicochemical programming of GelMA: tuning pore architecture, crosslinking/viscoelasticity, and channelized microstructures provides structural and mechanical cues for endothelial invasion, vascular network formation, and bone ingrowth. (**B**). The use of GelMA as a delivery matrix for cells/exosomes, growth factors, nucleic acids, ions, and nanoparticles to sustain pro-angiogenic and osteogenic cues. (**C**). Pathological versus physiological coupling: aging/critical defects show reduced type H vessels (CD31^hi/Emcn^hi), heightened ROS/inflammation, and impaired osteogenesis, whereas physiological coupling relies on type H vessels with Osx+ progenitors to support bone formation. Upward arrows (↑) indicate enhanced expression, activity, or biological outcomes, whereas downward arrows (↓) indicate reduced expression, activity, or biological outcomes. (**D**). Independent effects of GelMA structural tuning and exogenous cargo loading on vascularization and bone regeneration in fractures/defects. (**E**). Stimuli-responsive design (ROS, acidity, mechanics, photothermal inputs) enables on-demand degradation or cargo activation/release to match stage-specific vascularized osteogenesis. (**F**). Key challenges include dosing/kinetic control, such as burst release and insufficient tissue exposure, as well as unstable multi-factor interactions. In panel F, blue and red dots represent two different exogenous cargos loaded into GelMA. The equal signs indicate that the three GelMA systems contain equal amounts of the two cargos, whereas the unequal signs indicate divergent vascularization outcomes under equal-loading conditions, including synergistic enhancement, no additional synergistic increase, or antagonistic reduction of vascularization. Created in BioRender. Hu, C.Y.J. (2026) Available online: https://BioRender.com/5wf3o43 (accessed on 21 May 2026).

Medical-grade natural polymers are generally classified into two major categories: polysaccharides and their derivatives, and protein-/peptide-based materials [19]. These materials have been extensively used as scaffolds and delivery systems in tissue engineering. Gelatin methacryloyl (GelMA) is a photo-crosslinkable hydrogel obtained by methacryloylating gelatin chains [20,21], which combines the biological merits of gelatin with programmable processability. It retains native cell-adhesive motifs and enzyme-responsive degradability, thereby providing an ECM-mimetic microenvironment, while also enabling the fabrication of precisely defined microarchitectures via photopolymerization and 3D printing. As a result, GelMA has emerged as an attractive base material for vascularized bone tissue engineering [22,23]. Importantly, the key properties of GelMA can be systematically tuned by formulation parameters such as polymer concentration, degree of methacrylation, and crosslinking density, enabling angiogenic–osteogenic coupling to be induced through the material’s intrinsic physicochemical cues [24,25,26]. Accordingly, the overall GelMA-based strategy map, including intrinsic physicochemical programming, exogenous cargo loading, stimuli-responsive regulation, pathological coupling deficits, and key translational challenges, is schematically summarized in Figure 1.

Evidence from GelMA-based studies remains uneven. Many studies rely on in vitro tube formation, osteogenic markers, or short-term small-animal models, while long-term vascular function, bone quality, mechanical recovery, and safety are less often assessed. Key trade-offs also persist: high pro-angiogenic factor doses may promote early vessel formation but cause abnormal vascular structures or disrupt osteogenic timing; stronger crosslinking improves stability but may limit cell infiltration, vascular ingrowth, and degradation; and stimuli-responsive release systems do not always perform consistently in vitro and in vivo. Meanwhile, different strategies show distinct limitations. Structural and mechanical regulation offers better engineering controllability, but its ability to maintain mechanical support and rebuild perfusion in complex bone defects remains to be validated. Delivery of exogenous factors, cells, or extracellular vesicles provides strong biological stimulation, yet still faces challenges in dose control, release timing, safety, and standardization. Smart responsive systems may enable on-demand regulation in theory, but most remain at the proof-of-concept stage, with in vivo stability and manufacturing complexity limiting clinical translation.

This review focuses on three key points: how GelMA-based hydrogels regulate angiogenesis, osteogenesis, and their coupling; what levels of experimental and preclinical evidence support different strategies; and which approaches show greater translational potential in terms of reproducibility, mechanical adaptability, biosafety, manufacturability, and clinical feasibility, as well as the key barriers that remain.

**Table 1:** Biological cues and design implications in angiogenic–osteogenic coupling for GelMA systems.

Regulatory Axis	Key Biological Role	Design Implication	References
Vascular subtype specification and osteogenic niche	Type H vessels spatially co-localize with osteoprogenitors/osteoblasts Type H	Functional microvascular niche optimization	[4,5]
Temporal coupling between vascular invasion and ossification	Vascular invasion coordinates primary ossification	Early vascularization and temporal regulation	[27,28]
Osteoprogenitor–vascular feedback	Osteoprogenitors guide vascular patterning	Spatial vascular–osteogenic coordination	[6,7,8]
Hypoxia–HIF-1α/VEGF axis	Hypoxia-driven angiogenesis and vascularized repair	Perfusable vascular network formation	[29,30]
Blood flow/perfusion	Flow-dependent vascular function and nutrient transport	Perfusable and stable microvascular architecture	[9,10]
Endothelial Dll4/Notch signaling	Endothelial maturation and angiocrine regulation	Functional vascular maturation	[9,10,11]
VEGF–VEGFR signaling	Angiogenic–osteogenic coordination	Functional vessel formation	[12,13,14,15]
Aged endothelial secretome remodeling	Pro-osteogenic endothelial signaling decline	Restoration of endothelial niche signaling	[16,17]
Immune regulation of vascular–osteogenic coupling	Inflammation-resolving macrophage transition	Immunomodulatory vascular maturation	[31,32]

## 2. Mechanistic Review

To synthesize evidence on GelMA-based hydrogels for vascularized bone regeneration, this review used a structured search and stepwise screening approach. Studies related to GelMA and bone regeneration were identified, screened according to predefined criteria, and then classified by material design strategy, functional outcomes, and evidence type for further comparison and evaluation. The Web of Science Core Collection and PubMed were searched using terms related to GelMA-based materials, bone regeneration, and angiogenesis: (“GelMA” OR “gelatin methacryloyl” OR “gelatin methacrylate”) AND (“bone regeneration” OR “bone repair” OR “bone defect” OR osteogenesis OR osteogenic) AND (angiogenesis OR vascularization OR vascularisation OR vascularized OR vascularised OR endothelial OR “blood vessel” OR “type H vessel” OR CD31 OR endomucin OR EMCN). This search yielded 279 records from Web of Science and 205 from PubMed. Titles and abstracts were then screened to identify studies on GelMA-mediated vascularized bone regeneration.

During full-text screening, original studies were included if they focused on bone regeneration, bone defect repair, osteogenic differentiation, or vascularized bone regeneration; used GelMA or GelMA-based composite hydrogels as the main material platform; and reported defined strategies for regulating vascularization, osteogenesis, or angiogenic–osteogenic coupling. These strategies included structural/mechanical modulation, chemical modification, factor delivery, cell or extracellular vesicle delivery, inorganic ion/nanoparticle incorporation, and stimuli-responsive regulation. Studies also needed to include angiogenic or osteogenic assessments, such as VEGF, CD31, EMCN, HUVEC tube formation, ALP, ARS, RUNX2, OCN, micro-CT, or histological analysis.

Studies were excluded if they focused on non-bone tissues, used GelMA only as an auxiliary component, lacked angiogenic or osteogenic validation, or were reviews, editorials, conference abstracts, patents, or reports with incomplete experimental information. Studies based only on short-term in vitro results, with limited mechanistic analysis or translational evaluation, were not considered core evidence for comparative analysis.

As this review focuses on evidence strength and translational potential, the included studies were not regarded as equivalent. Priority was given to studies with both angiogenic and osteogenic evidence, animal model validation, load-bearing or near-load-bearing bone defects, longer follow-up, and information on mechanical performance, biosafety, or translational feasibility.

The included studies were further grouped into three strategies: intrinsic material programming, exogenous bioactive delivery, and stimuli-responsive/spatiotemporal regulation. They were compared in terms of mechanisms, evidence level, reproducibility, translational feasibility, and clinical limitations. Evidence level was judged by in vitro validation, animal model type, defect relevance, follow-up duration, and whether mechanical or biosafety assessments were included. Representative studies and their evidence features are summarized in Table 2, with more detailed comparative information provided in Supplementary Table S2.

**Table 2:** Representative GelMA-based strategies for vascularized bone regeneration, corresponding evidence/models, and major translational limitations.

Strategy	Representative Design Logic	Evidence/Model	Main Angiogenic–Osteogenic Evidence	Key Translational Limitation	References
Intrinsic spatial programming	Bilayer or osteon-like GelMA architecture	In vitro; rat cranial/large-defect models	Enhanced neurovascularization, angiogenesis, and osteogenesis	Non-load-bearing evidence; cell/multicomponent complexity	[33,34]
Intrinsic channel programming	Prevascular channels/hollow GelMA structures	In vitro; rat cranial defects	Improved vascular ingrowth and bone repair	Non-load-bearing evidence; limited large-animal validation	[35,36]
Intrinsic dynamic programming	VEGF-retaining dynamic GelMA network	Rat cranial defect	Sustained vascularization and osteogenesis	Nucleic acid stability; manufacturing complexity	[37]
Exogenous bioactive delivery	QK, Sr, Zn, or Si/P-loaded GelMA systems	In vitro; rat/osteoporotic/femoral defects	Enhanced angiogenesis and osteogenesis	Long-term safety; large-animal evidence lacking	[38,39,40]
Exogenous bioactive delivery	VEGF-, BMP-2-, DBM-, or vECM-GelMA systems	In vitro; rat/rabbit cranial defects	Enhanced vascularized bone formation	Dose, source, and batch variability	[41,42]
Exogenous bioactive delivery	Li-bioglass or DMOG-GelMA systems	Diabetic/high-glucose; femoral defects	Improved angiogenesis and osteogenesis under pathological conditions	Long-term safety and component variability	[43,44]
Exogenous bioactive delivery	PRP-GelMA within PCL framework	In vitro; rat femoral defect	Improved vascularized femoral repair	PRP variability; printing complexity	[45]
Spatiotemporal regulation	Timed VEGF/BMP-2-related delivery	In vitro; rat cranial defects	Improved stage-matched vascularized bone repair	Release control and scalable manufacturing	[46,47,48]
Spatiotemporal regulation	Cell-laden GelMA with mineral microspheres	In vitro; ectopic; mouse cranial defect	Improved perfusion and bone matrix deposition	Cellular complexity; GMP and scale-up barriers	[49]
Spatiotemporal regulation	Piezoelectric scaffold with Met-loaded GelMA	In vitro; rabbit femoral and rat calvarial defects	Sequential angiogenesis–osteogenesis coupling	Ultrasound/stimulation control; complex fabrication	[50]

Current GelMA-based studies show clear imbalance in evidence level and translational relevance. Exogenous bioactive delivery remains the dominant strategy, with the largest body of evidence and relatively rich in vivo validation, including diabetic bone defects, femoral condyle defects, and rabbit cranial defects. These systems often improve short-term outcomes such as CD31, VEGF, BV/TV, and new bone formation. However, their reliance on growth factors, extracellular vesicles, PRP, or multiple functional nanocomponents raises unresolved concerns regarding long-term safety, dose standardization, batch consistency, and GMP-compatible manufacturing.

Intrinsic material programming regulates vascular ingrowth and bone formation through pore architecture, microchannels, dynamic networks, and spatial organization. Although less extensively studied, this strategy relies less on exogenous bioactive components and may therefore offer advantages in manufacturing consistency, scale-up, and storage stability. However, most evidence still comes from in vitro studies and small-animal non-load-bearing defects, with limited assessment of functional perfusion, long-term vessel maturation, and load-bearing adaptation.

Stimuli-responsive and spatiotemporal regulation systems are closest to the dynamic nature of angiogenic–osteogenic coupling. Sequential release, ROS/pH-responsive delivery, piezoelectric regulation, and multistage delivery have been used to coordinate early vascularization with later osteogenic maturation and to mimic microenvironmental changes during bone repair. However, these systems often involve multiple components, complex release kinetics, and external stimulation parameters. Most remain at the small-animal or proof-of-concept stage, with long-term stability, parameter standardization, and clinical operability still insufficiently validated. The following sections therefore compare the three GelMA-based strategies in terms of mechanism, evidence level, reproducibility, translational feasibility, and clinical limitations.

## 3. Intrinsic Material Programming

Impaired bone regeneration is commonly driven by insufficient perfusion, reduced endothelial responsiveness, and chronic inflammation/oxidative stress, which together compromise angiogenic–osteogenic coupling. Intrinsic material programming regulates cell behavior by tuning the structural and physicochemical properties of GelMA hydrogels, including composition, geometry, permeability, and mechanical properties, thereby improving stability and reproducibility without relying on exogenous factors [51]. However, such strategies remain highly dependent on intrinsic material properties and therefore have limited adaptability to dynamic microenvironmental changes. This section discusses factor-free GelMA strategies across structural, mechanical, and interfacial dimensions, focusing on their roles and limitations in promoting angiogenesis and vascular remodeling.

### 3.1. Spatial Architecture: Promoting Early Vascular Ingrowth

**Figure 2:**
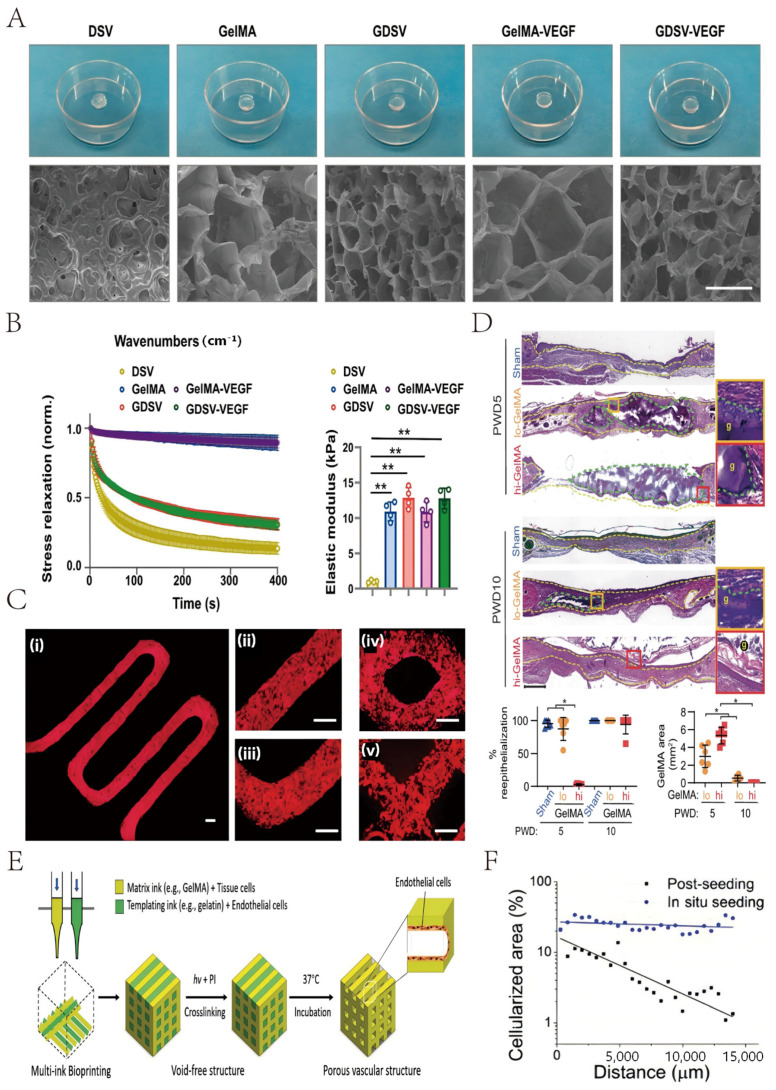
Spatial architecture and mechanical properties of GelMA hydrogels. (**A**). Macroscopic appearance and SEM images of hydrogels with different formulations (DSV, GelMA, GDSV, GelMA–VEGF, and GDSV–VEGF), highlighting differences in pore size and network interconnectivity (scale bar: 100 µm). Reprinted from Ref. [37]. (**B**). Normalized stress relaxation behavior and elastic modulus of the hydrogels, illustrating how matrix stiffness and viscoelastic dissipation jointly support endothelial rearrangement and 3D network stabilization. Statistical significance: two asterisks indicate *p* < 0.01, respectively. Reprinted from Ref. [37]. (**C**). Luminal endothelialization of RFP-labeled HUVECs in perfused microchannels: (**i**–**iii**) S-shaped single channel after 12 days; (**iv**,**v**) circular joint and lattice channels after 10 days. Reprinted from Ref. [72]. (**D**). H&E staining of GelMA implants with different crosslinking degrees at PWD5 and PWD10. Yellow and red boxes indicate regions shown at higher magnification on the right; yellow dashed lines indicate tissue-layer boundaries; green dashed lines mark the GelMA–tissue interface; and “g” denotes the GelMA region. Quantification of the re-epithelialization ratio and residual material area is shown. Asterisks above brackets indicate statistically significant differences between the indicated groups, Re-epithelialization: *p* < 0.0001; GelMA area: PWD5 lo/hi: *p* = 0.0003, lo-GelMA PWD5/10: *p* = 0.0002, hi-GelMA PWD5/10: *p* < 0.0001. Reprinted from Ref. [57]. (**E**). Schematic of sacrificial template 3D printing (VF-3DP) for constructing fully interconnected microchannels. Reprinted from Ref. [72]. (**F**). Quantification of endothelial coverage uniformity within microchannels; the cellularized area (fluorescence coverage) is used to assess luminal endothelialization. Reprinted from Ref. [72].

Within biomaterial matrices, early vascular ingrowth is constrained by pore size, interconnectivity, and pore throat dimensions, which govern endothelial infiltration, network connectivity, perfusion establishment, and vascular maturation [52,53,54]. Porous GelMA cryogel microspheres enable pore size regulation via gradient-freezing protocols. When the mean pore diameter is close to 15 µm, BMSC and HUVEC adhesion/proliferation, osteogenic/angiogenic responses, and CD31/OCN expression after implantation are enhanced [55]. Low-concentration GelMA hydrogels (5% *w*/*v*) also form larger pores, facilitating intra-scaffold diffusion and BMSC osteogenic differentiation in vitro [56]. Crosslinking degree and stiffness further reshape pore architecture and cellular infiltration depth, thereby modulating host immune responses. Highly crosslinked, stiff matrices promote pro-inflammatory macrophage accumulation, reactive oxygen species, foreign-body giant cells, fibrotic encapsulation, and poor integration, whereas lightly crosslinked matrices facilitate cell infiltration, degradation, and constructive repair (Figure 2D) [57,58]. Denser networks restrict cell migration and 3D anastomosis, while open interconnected porosity favors infiltration and vascular maturation [59]. However, pore architecture optimization involves clear trade-offs: larger pores and higher interconnectivity facilitate vascular ingrowth but may compromise mechanical support, whereas compact structures enhance mechanics but restrict infiltration and vascular formation. Spatially partitioned designs can partially decouple these functions. For example, two-step photopatterning creates low-concentration “vascular zones” and high-concentration “osteogenic zones” within GelMA, producing organized vessels around a mineralized core and enhancing both vascular organization and osteogenic marker expression [60]. However, the fabrication complexity, reproducibility, and translational potential of such heterogeneous designs remain to be validated.

### 3.2. Mechanical Properties: Enabling Endothelial Rearrangement and Anastomosis to Stabilize Network Connectivity

During hydrogel-guided angiogenesis, matrix stiffness must fall within an optimal window to allow early sprouting to evolve into a continuous 3D vascular network. An overly soft matrix is prone to collapse and pore closure, whereas an excessively stiff matrix restricts cellular traction, migration, and rearrangement, thereby limiting vascular extension and anastomosis [61,62]. However, maintaining this stiffness window in vivo remains challenging because material degradation, cellular remodeling, and local mechanical evolution continuously shift the mechanical microenvironment. Mechanotransduction is also central to bone regeneration, and tuning scaffold stiffness and viscoelasticity can regulate osteoblast function and repair efficiency [63]. Increasing the elastic modulus of alginate–GelMA matrices enhances osteogenesis by promoting YAP nuclear translocation, biomolecular condensation, osteogenic marker expression, and mineralization; in vivo, modulus optimization further increases bone formation [64]. Stress relaxation provides an additional mechanics-based strategy to balance structural support with adaptive deformation during angiogenesis. For example, introducing dynamic covalent bonds into a gelatin-based photo-crosslinked network yields a double-network dynamic bioink that maintains printing fidelity while exhibiting accelerated relaxation, thereby promoting endothelial self-assembly, 3D network connectivity, vascular network formation, and tissue integration [65]. To emulate native bone ECM dynamics, a GelMA/DNA double-network hydrogel combines a stable GelMA framework with a reversible DNA network, providing dynamic viscoelasticity and programmable stress relaxation. Functionalization with Apt19S and AptV further enables recruitment of osteogenic cells and endothelial cells (Figure 2A,B) [37]. Zhu et al. similarly constructed a DNA–GelMA dynamic hydrogel that maintains mechanical integrity while promoting 3D cell migration, self-organization, bone-like tissue formation, osseointegration, endothelial function, and angiogenesis-related marker expression [66]. However, although dynamic crosslinking and multifunctional modules enable precise cell regulation in experimental models, increased system complexity imposes higher demands on fabrication stability, batch-to-batch consistency, and in vivo controllability.

### 3.3. Organized Microstructures: Using Alignment and Channels to Improve Network Formation and Perfusion Accessibility

Anisotropic fiber alignment can direct endothelial migration and elongation, promote junctional remodeling, and improve endothelial monolayer integrity. For example, Du et al. fabricated a co-electrospun PCL/GelMA nanofiber lining on the inner surface of a shape-memory tubular scaffold, enhancing endothelial adhesion, elongation, and VE-cadherin-mediated junction formation [67]. Preformed microchannels provide structural guidance for intrahydrogel vascularization, shifting the process from passive cell infiltration to rapid, directed endothelialization and earlier perfusion establishment. Such channels can be generated via sacrificial templating, printing, microfluidics, or directional freezing, reducing transport barriers and improving network interconnection [68,69,70]. Compared with formulation-based regulation, organized microstructures are dictated primarily by fabrication: extrusion/printing generates alignment and interlayer pathways, whereas sacrificial templating or molding enables designed, interconnected channel networks [71]. Compared with formulation-based regulation, organized microstructures are dictated primarily by fabrication: extrusion/printing generates alignment and interlayer pathways, whereas sacrificial templating or molding enables designed, interconnected channel networks [60]. Stevens and co-workers used thermoresponsive gelatin as a sacrificial template and dual-nozzle 3D printing to fabricate a pore-free GelMA construct with an interconnected 3D channel network. After gelatin dissolution, perfusable channels formed and enabled in situ HUVEC endothelialization, producing a continuous CD31^+^ endothelial monolayer and stable perfusion under microfluidic flow (Figure 2C,E,F) [72]. However, structure-guided strategies still face notable in vivo limitations. Predefined anisotropic architectures or microchannels may struggle to adapt to dynamic tissue remodeling, and their long-term stability and integration with host vasculature remain uncertain. Although preformed channels can accelerate initial perfusion, whether they can evolve into fully functional vascular networks remains unclear. Advanced fabrication techniques enable precise structural control in experimental models, but their complexity, reproducibility in large-scale defects, and translational feasibility remain significant challenges.

### 3.4. Chemical Features

**Figure 3:**
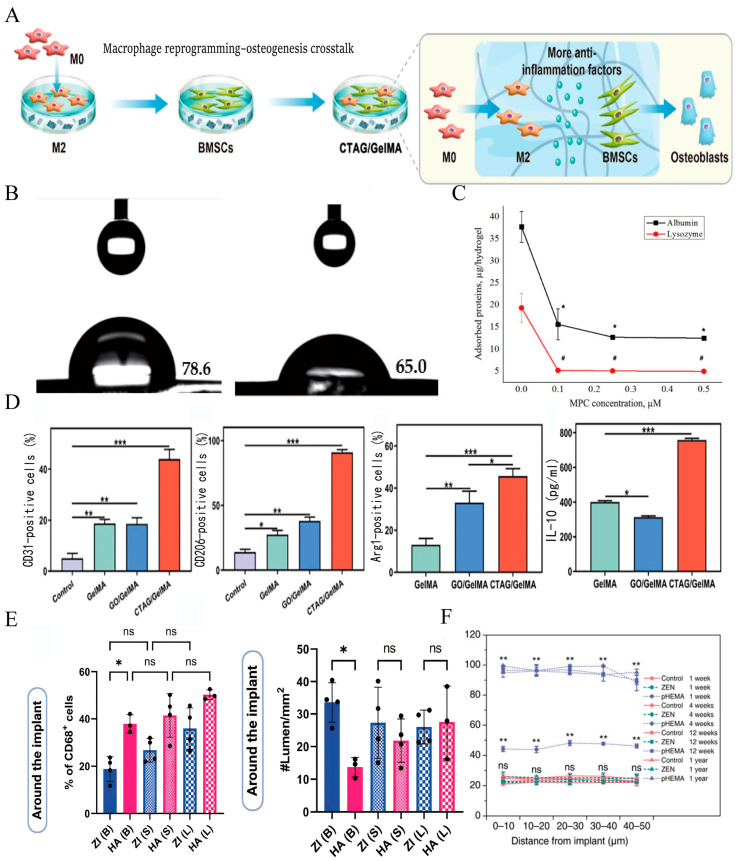
Chemical interfacial functionalization of GelMA modulates protein adsorption. (**A**). Schematic of macrophage reprogramming coupled to osteogenesis. Interfacial functionalization modulates protein adsorption and inflammatory cues, promotes M2 polarization, and drives BMSC osteogenic differentiation. Reprinted from Ref. [79]. (**B**). Water contact angle-based wettability of MPC-functionalized surfaces, showing enhanced hydrophilicity and a strengthened hydration layer that modulates protein adsorption. Reprinted from Ref. [73]. (**C**). Protein adsorption on functionalized surfaces, showing reduced albumin/lysozyme binding and altered adsorption profiles that reshape early immune cues. Reprinted from Ref. [73]. Statistical significance: asterisks and hash symbols indicate *p* < 0.05 versus the control group. (**D**). Upregulation of CD206/Arg-1, IL-10, and CD31, indicating M2-skewed immunomodulation and enhanced angiogenic support. Statistical significance: one, two, and three asterisks indicate *p* < 0.05, *p* < 0.01, and *p* < 0.001, respectively. Reproduced from Ref. [79]. (**E**). Peri-implant inflammation and lumen formation. Quantification of CD68^+^ cells and vessel lumen number per area shows reduced inflammatory cell accumulation and increased vascular structure density after interface modification. Statistical significance: *ns*, not significant; asterisk indicate *p* < 0.05, respectively. Reprinted from Ref. [74]. (**F**). Peri-implant collagen deposition over time, showing reduced fibrotic encapsulation and improved long-term tissue integration after interfacial optimization. Statistical significance: *ns*, not significant; two asterisks indicate *p* < 0.01, respectively. Reprinted from Ref. [78].

After implantation, cells primarily interact with the rapidly formed protein adsorption layer on the material surface rather than the bulk matrix itself. Strongly hydrated zwitterionic groups, such as phosphorylcholine, stabilize the interfacial hydration layer and suppress protein adsorption. For example, p(MPC) modification reduces surface contact angle and adsorption of serum proteins such as albumin and lysozyme, thereby modulating early immune recognition (Figure 3B,C) [73]. The composition and conformation of the adsorbed layer further influence immune activation, endothelial migration, vascular maturation, and osteoblast adhesion/differentiation. In vivo, low-protein-adsorption interfaces reduce fibrotic responses and collagen encapsulation while improving tissue integration and vascular density (Figure 3E) [74]. Accordingly, chemical interfacial functionalization of GelMA can regulate protein adsorption, adhesion signaling, and immune microenvironmental responses without relying on exogenous factor release. GAG- or heparin-mimetic modifications reshape interfacial protein presentation and support subsequent cell adhesion and tissue remodeling [75]. Covalently anchoring functional motifs within GelMA enables stable bioactive cue presentation while avoiding burst release and molecular deactivation. For example, Qiao et al. co-crosslinked an osteogenic peptide with GelMA, enhancing osteogenic gene expression, calcium deposition, and mineralization in vitro and in vivo [76]. Covalent incorporation of VEGF-mimetic peptides similarly enhances endothelial migration and tubular network formation without relying on release (Figure 3F) [77,78]. Recent studies further show that chemical modification of GelMA-based composites reshapes interfacial biological responses and enhances angiogenesis and bone regeneration (Figure 3A,D) [79,80,81]. However, non-release strategies still present limitations. Excessive suppression of protein adsorption may impair cell adhesion and signaling recognition, whereas covalently immobilized ligands lack dynamic regulation and temporal adaptability. The main intrinsic material programming strategies are summarized in Table 3, and detailed mechanistic effects on angiogenic–osteogenic coupling are provided in Supplementary Table S3.

## 4. Exogenous Bioactive Delivery

The intrinsic physicochemical properties of hydrogels, including pore interconnectivity, mechanical support, permeability, and remodelability, provide the structural basis for vascular ingrowth and osteogenesis. However, in aging, ischemic/inflammatory, and critical-sized defects, intrinsic signaling alone is often insufficient to rapidly establish a stable, perfusable microvascular network [82,83,84]. Therefore, exogenous bioactive cues are frequently incorporated to enhance vascularization and osteogenesis [85,86,87]. Although combined cue delivery may improve synergy, dosing remains difficult to control, creating a trade-off between suppressing burst release and maintaining sufficient exposure [88,89]. In addition, mechanisms underlying multi-cue combinations are often incompletely defined, and increased complexity may introduce signaling cross-talk and antagonistic interactions [90].

### 4.1. Cells and Vesicles

**Figure 4:**
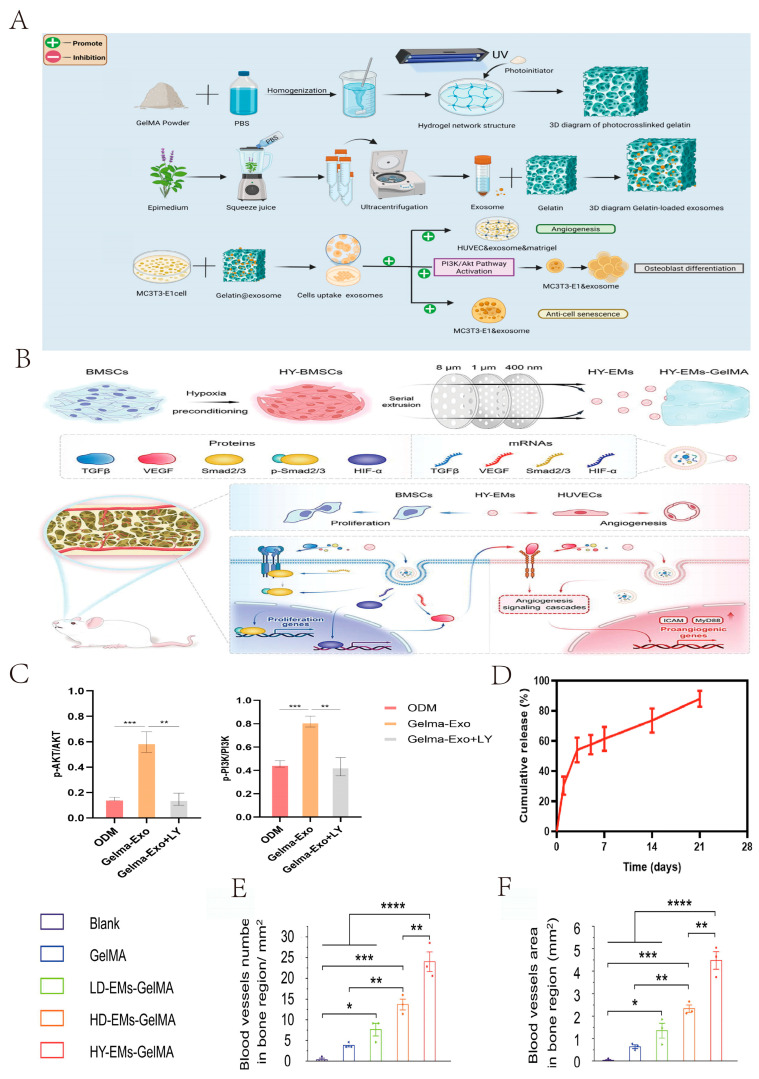
GelMA hydrogels synergize with exogenous bioactive cues to promote vascularized osteogenesis. (**A**). Plant-derived exosomes encapsulated in GelMA for local delivery; enhanced cellular uptake and paracrine modulation support osteogenic cell responses and initiate pro-angiogenic signaling. Reprinted from Ref. [98]. (**B**). Hypoxia-preconditioned BMSC-derived exosome mimetics (HY-EMs) loaded in GelMA to reinforce pro-angiogenic and pro-osteogenic paracrine cues, coordinating neovascularization and bone formation in defects. Reprinted from Ref. [97]. (**C**). Quantitative evidence of PI3K/Akt activation. The p-PI3K/PI3K and p-Akt/Akt ratios indicate enhanced PI3K/Akt signaling in GelMA@Exo, which is attenuated by the inhibitor LY294002, supporting pathway involvement. Statistical significance: two and three asterisks indicate *p* < 0.01 and *p* < 0.001, respectively. Reprinted from Ref. [98]. (**D**). Example of VEGF exposure kinetics in GelMA systems. In GelMA@Ly-PRF, VEGF shows early elevated exposure followed by sustained levels over time. Reprinted from Ref. [102]. (**E**). Quantification of neovessel numbers in defect sites. Exogenous-cue-synergized GelMA significantly increases vessel counts versus controls. Statistical significance: one, two, three, and four asterisks indicate *p* < 0.05, *p* < 0.01, *p* < 0.001, and *p* < 0.0001, respectively. Reprinted from Ref. [97]. (**F**). Quantification of vascular area in defect sites. Vascular area is expanded by the synergistic system, supporting a more stable and perfusable vascular network. Statistical significance: one, two, three, and four asterisks indicate *p* < 0.05, *p* < 0.01, *p* < 0.001, and *p* < 0.0001, respectively. Reprinted from Ref. [97].

In GelMA-based delivery systems, the therapeutic efficacy of cells and vesicles depends not only on loading dose but also on their ability to retain functionality within ischemic microenvironments. GelMA’s 3D network prolongs local retention of bioactive cargos and enables controllable release, thereby stabilizing pro-angiogenic and pro-osteogenic signaling [91,92,93,94,95]. Liu et al. developed injectable phosphorylated GelMA microspheres loaded with bone marrow mesenchymal stem cells. By strengthening cell–cell interactions and matrix adhesion, this platform improved transplanted cell survival and synergistically enhanced angiogenesis and osteogenesis in a calvarial defect model [96]. PGMSs co-loaded with a BMSC–HUVEC co-culture similarly accelerated neovascularization and bone formation in rat calvarial defects [58]. In studies using exosome-mimetic HY-EMs, GelMA prolonged local retention and sustained release, thereby enhancing vascularized bone regeneration (Figure 4B,E,F) [97]. Another study engineered a GelMA hydrogel loaded with Epimedium-derived exosomes, where sustained release promoted osteoblast proliferation, mineralization, and osteogenic marker expression through PI3K/Akt activation (Figure 4A,C) [98]. However, cell- and vesicle-based delivery strategies face intrinsic limitations. Cell survival and long-term functionality remain unstable in ischemic and inflammatory environments, whereas exosomes and their mimetics exhibit substantial heterogeneity and poor standardization. In addition, most evidence is still derived from small, non-load-bearing animal models.

### 4.2. Delivery of Biomacromolecules

Biomacromolecules provide potent cues for vascularized bone regeneration, yet their major limitation lies in rapid loss, short half-life, and instability within hostile microenvironments [99,100,101]. Accordingly, GelMA-based delivery platforms are designed to prolong signal retention and release. Encapsulating lyophilized platelet-rich fibrin within GelMA enables sustained release of factors such as VEGF for up to 21 days, enhancing endothelial angiogenic responses and bone regeneration in a rat calvarial defect model (Figure 3D) [102]. Another study physically loaded VEGF into a GelMA/DNA double-network hydrogel, achieving sustained release (~70% over 14 days) while synergizing with dynamic matrix mechanics and aptamer-mediated cell targeting to promote neovessel formation and bone regeneration [37]. Click chemistry has also been used to covalently graft a dual osteogenic/angiogenic peptide onto GelMA microspheres, establishing a long-acting peptide delivery system that simultaneously activated osteogenic and angiogenic signaling pathways and accelerated bone defect repair [103]. However, although controlled-release strategies prolong signal exposure, their release profiles remain largely predefined by degradation or diffusion and are highly susceptible to in vivo conditions. Sustained release therefore lacks stage-specific and microenvironment-responsive regulation, limiting long-term precision in bone repair.

### 4.3. Inorganic Ions and Nanoscale Delivery

The use of inorganic ions and nanomaterials in GelMA-based delivery systems has expanded rapidly in recent years. Owing to their physicochemical stability and tunable interactions with polymer networks, these components can achieve long-term local retention and controllable release within hydrogels, offering a materials-centric route through which to regulate angiogenesis and osteogenesis through relatively simple formulation designs [25,33,104,105]. Liu et al. loaded Ce^3+^ into porous phosphorylated GelMA microspheres to achieve sustained ion release. Ce^3+^ not only directly enhances osteogenic differentiation of bone marrow mesenchymal stem cells but also upregulates pro-angiogenic factors such as VEGF, thereby improving endothelial tube formation. In an animal bone defect model, combining Ce^3+^-releasing microspheres with BMSCs markedly promoted both new bone formation and vascular network development, highlighting the potential of ion–cell synergy for regeneration [96]. A nucleic acid delivery system has been developed by encapsulating miR-21-5p-loaded tetrahedral DNA nanostructures (TDNs) within GelMA hydrogels. In vivo, the composite scaffold enables sustained release of the active cargo, significantly enhancing angiogenesis and osteogenic differentiation in the defect region and thereby promoting calvarial regeneration [106]. One study engineered a GelMA composite hydrogel (GCPM) co-loaded with CeO_2_@PDA nanoparticles and Mg^2+^. By scavenging ROS and modulating immune responses via the nanoparticles, together with the pro-angiogenic activity of Mg^2+^, the system synergistically improved the pathological microenvironment of osteoporotic bone defects. In animal models, GCPM markedly enhanced both neobone formation and vascularization [107]. Despite their advantages, inorganic ions and nanomaterials face inherent challenges in in vivo control. Release is largely passive and difficult to precisely regulate, raising risks of local fluctuation and accumulation. Long-term biosafety remains uncertain, particularly regarding cytotoxicity and immune responses. Furthermore, their multi-pathway effects, while potentially synergistic, introduce significant mechanistic complexity and variability across microenvironments. Achieving functional enhancement with predictable and controllable outcomes therefore remains a key challenge.

## 5. Stimuli-Responsive and Temporally Programmed Systems

**Figure 5:**
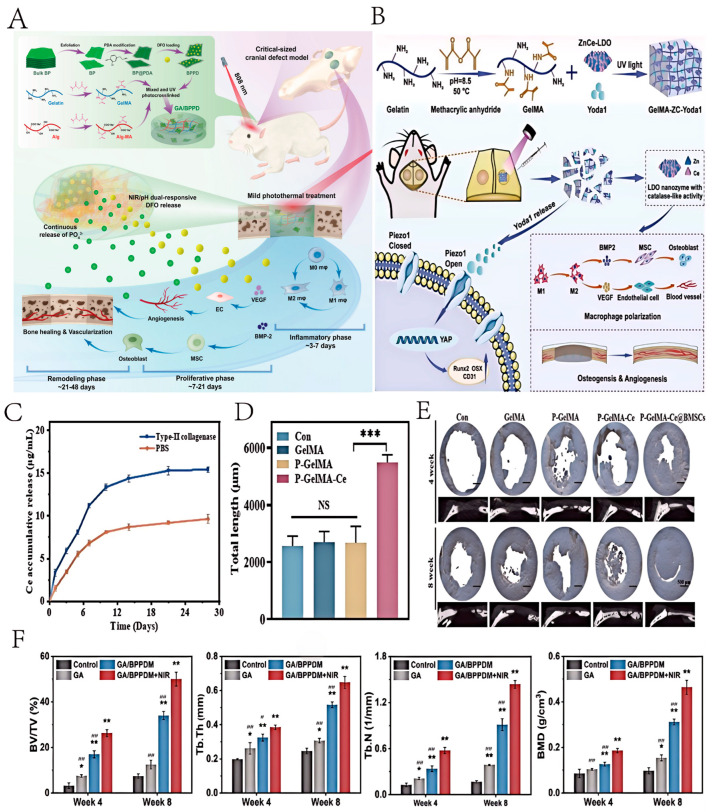
Inorganic ion/nanocomponent delivery and stimuli-responsive GelMA systems for vascularized bone regeneration. (**A**). Schematic of an NIR/pH dual-responsive GelMA platform. Mild photothermal heating and acidic activation enable ROS scavenging, immunomodulation, and on-demand release of pro-angiogenic/osteogenic cues for stage-adaptive vascularized bone. Reprinted from Ref. [114]. (**B**). Schematic of GelMA–ZC–Yoda1, showing Zn/Ce nanozyme-mediated H_2_O_2_ scavenging and pro-repair macrophage polarization, combined with sustained Piezo1 activation, which couples angiogenesis with mechano-osteogenic signaling. Reprinted from Ref. [116]. (**C**). Ce^3+^ release from P-GelMA microspheres. In vitro release profiles under different conditions show prolonged local retention and gradual release within the gel network, enabling long-acting regulation of osteogenic and angiogenic cues. Reprinted from Ref. [96]. (**D**). Quantification of HUVEC tube formation. Total tube length indicates that ion/nanocomponent-containing GelMA systems significantly enhance angiogenic capacity. Statistical significance: NS, not significant; three asterisks indicate *p* < 0.001. Reprinted from Ref. [96]. (**E**). Mineralization in calvarial defects assessed by micro-CT, which, at 4 and 8 weeks, shows increased mineralization and new bone formation with nanocomponent/ion delivery. Reprinted from Ref. [96]. (**F**). Quantification of bone microarchitecture. BV/TV, Tb.Th, Tb.N, and BMD demonstrate enhanced bone formation and structural reconstruction in vivo with composite nano/ion delivery systems. Data are presented as the mean ± SD; *n* = 3. Statistical significance: one and two asterisks indicate *p* < 0.05 and *p* < 0.01 versus the control group, respectively; one and two hash symbols indicate *p* < 0.05 and *p* < 0.01 versus the GA/BPPDM+NIR group, respectively. Reprinted from Ref. [114].

Bone repair is not a single linear process. The early phase requires control of inflammation and oxidative stress, restoration of local perfusion, and promotion of angiogenesis, whereas the later phase depends more on vessel maturation, matrix deposition, mineralization, and osteogenic remodeling [108,109]. Conventional sustained-release systems are limited in meeting the stage-specific demands of bone repair. Although preset structural and chemical modulation of GelMA can promote angiogenic–osteogenic coupling by altering pore architecture, crosslinking density, and spatial heterogeneity, these systems cannot readily adapt to microenvironmental changes during repair. Stimuli-responsive and spatiotemporally regulated GelMA systems have therefore emerged to better match material behavior with stage-specific biological needs. In the first step, endogenous or exogenous triggers, including ROS, acidic pH, enzymatic activity, photothermal effects, ultrasound, or mechanical stimulation, enable on-demand release, degradation, or functional activation [110,111,112,113]. Second, spatial compartmentalization, differential degradation, or sequential release allows stage-specific delivery of pro-angiogenic signals in the early phase and pro-osteogenic signals in the later phase. Representative GelMA systems based on inorganic ion/nanocomponent delivery and stimuli-responsive regulation are summarized in Figure 5A–F, including NIR/pH-responsive release and immune regulation (Figure 5A), Zn/Ce nanozyme–Yoda1-mediated mechanochemical coupling (Figure 5B), Ce^3+^ release behavior and endothelial tube formation (Figure 5C,D), and micro-CT-based mineralization and bone microarchitectural reconstruction in vivo (Figure 5E,F).

In terms of stimuli-responsive regulation, NIR/pH dual-responsive GelMA composite hydrogels have been reported to scavenge ROS and promote M2 macrophage polarization under mild photothermal stimulation. Under acidic conditions, these systems can also control the release of DFO and PO_4_^3−^, thereby enhancing vascularization and bone regeneration in rat calvarial defects [114]. ROS-responsive GelMA hydrogels can undergo ROS-triggered degradation to release METRNL, thereby reducing oxidative stress and inflammation while activating the c-Kit/PI3K/Akt pathway to promote angiogenesis and recruit endogenous BMSCs [115]. Similarly, the GelMA–ZC–Yoda1 system integrates Zn/Ce nanozymes with the Piezo1 agonist Yoda1, enabling ROS scavenging, immune modulation, and mechanosensitive signal activation. These effects jointly regulate the inflammatory microenvironment and promote angiogenesis and osteogenesis [116].

For temporal programming, VEGF/GelMA can be combined with PCSK9/vascular-derived ECM composite hydrogels to achieve differential release, coupling early vascularization with later osteogenic differentiation [46]. Spatiotemporally graded GelMA bone organoids use rapid GelMA degradation to release DMOG for early prevascularization, while slower mineralized microsphere degradation supports later osteogenic maturation [49]. Related designs include GelMA-VEGF-filled PCL/nHA/Laponite scaffolds for sustained VEGF release [47], Janus HAMA/GelMA microcarriers for sequential VEGF/BMP-2 delivery [48], and BTO/nHA/PCL scaffolds with Met-loaded GelMA, which combine piezoelectric stimulation with drug release to coordinate early angiogenesis and later osteogenesis [50].

Compared with conventional sustained-release platforms, stimuli-responsive and spatiotemporally regulated GelMA systems better match the dynamic process of angiogenic–osteogenic coupling. However, their in vivo performance remains difficult to predict because temporal release depends on degradation and diffusion, while responsive mechanisms are affected by ROS levels, pH changes, tissue heterogeneity, stimulus penetration depth, and clinical operability. Multicomponent, multistage, and multi-stimulus designs further increase system complexity, batch variability, and engineering difficulty. Thus, balancing temporal control, reproducibility, and clinical applicability remains a key challenge for their translation.

## 6. Critical Synthesis of GelMA-Based Strategies for Angiogenic–Osteogenic Coupling

### 6.1. Mechanistic Comparison

GelMA-based strategies for vascularized bone regeneration can be grouped into intrinsic material programming, exogenous bioactive delivery, and stimuli-responsive/spatiotemporal regulation. Intrinsic material programming relies on pore architecture, microchannels, dynamic networks, anisotropic structures, and osteon-like spatial organization to guide vascular ingrowth and bone formation. It offers better structural controllability and manufacturing consistency, but its short-term biological stimulation is relatively limited. Exogenous bioactive delivery uses VEGF, BMP-2, PRP/CGF, extracellular vesicles, bioactive ions, or nanoparticles to directly promote endothelial activation, angiogenesis, and osteogenic differentiation, but remains limited by dose control, release kinetics, and long-term safety. Stimuli-responsive and spatiotemporally regulated systems further aim to coordinate early vascularization with later osteogenic maturation through ROS/pH responses, piezoelectric stimulation, adaptive physicochemical changes, or multistage delivery. These systems better mimic dynamic bone repair, but their multicomponent design and complex triggering conditions increase translational difficulty. Together, these strategies indicate a shift from enhancing single biological outcomes toward reconstructing dynamic vascularized bone repair.

### 6.2. Evidence-Level Comparison

Current GelMA-based studies for vascularized bone regeneration show clear imbalance in evidence level. Exogenous bioactive delivery has the richest preclinical evidence, with models extending from rat calvarial defects to osteoporotic femoral condyle defects [38], high-glucose or diabetic femoral defects [43], rat distal femoral/femoral condyle defects [39,44,45], and rabbit cranial or ectopic vascularized bone models [41]. Angiogenesis is usually evaluated by CD31, α-SMA, VEGF, or tube formation, while osteogenesis is assessed by micro-CT parameters, histology, and markers such as OCN, OPN, and RUNX2 [38,39,41,42,43,44,45]. Nevertheless, most studies remain limited to small- or medium-sized animals with short follow-up periods, usually 4–8 weeks or 1–3 months [39,41,42,43,44,45]. Evidence from clinically relevant load-bearing large-animal models, such as sheep, goats, pigs, or dogs, is still lacking, leaving an important gap in long-term and load-bearing validation.

The evidence for intrinsic material programming remains relatively limited, with most studies confined to in vitro experiments and small-animal non-load-bearing cranial defects observed over relatively short periods (typically 4–8 weeks). Representative designs include pore regulation, prevascular channels, dynamic networks, and osteon-like spatial organization [33,34,35,36,37]. These strategies can promote vascular ingrowth, vascular network formation, and bone repair, as shown by osteon-like dual-ring GelMA systems [34], dynamic GelMA networks [37], and hollow or prevascular channel structures assessed in 4/8-week rat skull defect models [35,36]. However, current evaluations mainly rely on CD31 expression, Microfil perfusion, or HUVEC tube formation, with limited evidence for long-term vessel maturation, stable perfusion, and true bone–vascular spatial organization [35,36,37]. Despite weaker preclinical validation, the lower dependence on exogenous components, better structural controllability, and stronger manufacturing consistency make intrinsic material programming a promising direction for engineering translation.

Stimuli-responsive and spatiotemporally regulated GelMA systems are mechanistically closer to the dynamic process of angiogenic–osteogenic coupling, but their evidence maturity remains limited. Representative studies include GelMA-VEGF-filled PCL/nHA/Laponite scaffolds tested in rat critical-sized calvarial defects at 6 weeks [47]; spatiotemporally graded GelMA bone organoids evaluated in mouse critical-sized calvarial defects at 4 and 8 weeks [49]; and BTO/nHA/PCL scaffolds combined with Met-loaded GelMA tested in rabbit femoral condyle defects at 6 and 12 weeks and rat calvarial defects at 4 and 8 weeks [50]. These systems better mimic the sequence of early vascularization followed by osteogenic maturation. However, the evidence still mainly comes from in vitro studies, small-animal cranial defects, or limited rabbit femoral condyle models, while large-animal load-bearing validation, longer follow-up, and clinical operability remain unclear. Therefore, they should be viewed as mechanistically advanced but early-stage proof-of-concept strategies.

### 6.3. Translational Comparison

Current GelMA-based studies for vascularized bone regeneration still lack high-level evidence that is truly close to clinical translation. Evidence maturity does not necessarily parallel mechanistic sophistication: exogenous delivery has the strongest in vivo evidence, intrinsic material programming appears more realistic for engineering translation, whereas stimuli-responsive and spatiotemporally regulated systems are mechanistically advanced but remain largely proof-of-concept. Therefore, the most promising systems at this stage are not those with the greatest functional complexity, but moderately complex GelMA composites with defined compositions, sufficient animal validation, stable angiogenic and osteogenic evidence, and a reasonable balance among manufacturing consistency, mechanical adaptability, and long-term safety.

## 7. Conclusions and Future Prospects

### 7.1. Conclusions

GelMA hydrogels have become one of the most representative material platforms for vascularized bone regeneration because of their biocompatibility, tunable mechanical properties, ECM-mimetic features, and compatibility with 3D biomanufacturing. Current studies have moved beyond simply promoting osteogenesis or angiogenesis toward multidimensional regulation of angiogenic–osteogenic coupling, mainly through intrinsic material programming, exogenous bioactive delivery, and stimuli-responsive or spatiotemporal regulation. Increasing attention has also been given to vascularization, immune regulation, microenvironmental remodeling, and multicellular interactions, suggesting that GelMA systems are evolving from passive scaffolds into dynamic regulatory platforms.

However, our reassessment of the current evidence shows clear imbalance in evidence level and translational readiness. Exogenous bioactive delivery has the richest preclinical evidence and strong short-term pro-angiogenic and osteogenic effects, but long-term safety, dose standardization, release predictability, and GMP quality control remain insufficiently addressed. Intrinsic material programming has a smaller evidence base, but its lower dependence on high-dose growth factors, living cells, or complex natural components gives it more realistic advantages in manufacturing consistency, sterilization compatibility, storage stability, and engineering scale-up. Stimuli-responsive and spatiotemporally regulated systems are closest to the dynamic process of angiogenic–osteogenic coupling, but most remain at the small-animal or proof-of-concept stage, and their complex components, external stimulation parameters, and multistage release behaviors increase the difficulty of standardization and clinical implementation.

Notably, systems with stronger evidence are not necessarily those with the most complex mechanisms. At this stage, moderately complex GelMA composites with defined compositions, controllable structures, printability or injectability, combined vascular regulation and mechanical support, and good manufacturing consistency may be closer to realistic clinical translation. Future work should therefore move away from simply adding functional modules or increasing mechanistic complexity, and instead focus on predictability, reproducibility, and clinical feasibility.

Based on the current evidence structure, the translation of GelMA-based vascularized bone regeneration systems can be viewed in three stages. In the short term, standardized evaluation systems should be established. In addition to commonly used markers such as CD31, VEGF, ALP, and BV/TV, future studies should assess functional perfusion, vessel maturation, long-term bone remodeling, degradation behavior, and mechanical stability. In the medium term, models should move beyond non-load-bearing small-animal defects toward pathological, load-bearing, or near-load-bearing bone defects, followed by long-term large-animal studies. In the long term, GMP manufacturing, sterilization, storage, and regulatory pathways must be considered in parallel. For the three major strategy types, exogenous delivery systems should prioritize dose windows and release kinetics; intrinsic material programming should strengthen manufacturability and mechanical adaptation; and stimuli-responsive systems should prove the reproducibility of response parameters and clinical operability.

**Figure 6:**
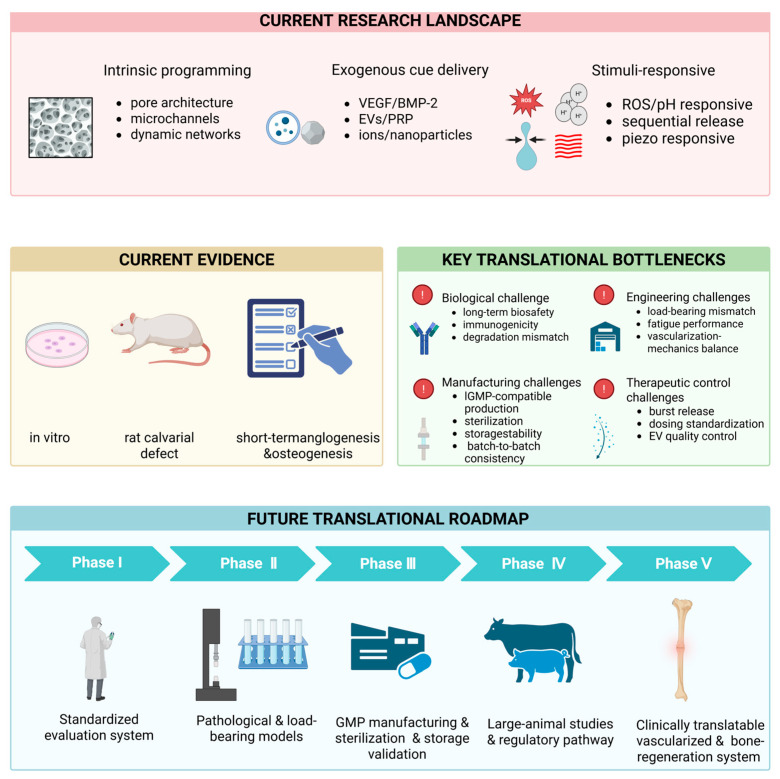
Current GelMA-based strategies, evidence structure, translational bottlenecks, and future roadmap for vascularized bone regeneration. Current studies are mainly based on intrinsic material programming, exogenous bioactive delivery, and stimuli-responsive/spatiotemporal regulation systems, but evidence still relies largely on in vitro and short-term small-animal models. Key translational challenges include biosafety, degradation matching, vascularization–mechanics balance, manufacturing consistency, and release controllability. Future translation should progressively advance toward standardized evaluation, pathological/load-bearing models, GMP-compatible manufacturing, large-animal validation, and clinically translatable vascularized bone regeneration systems. Created in BioRender Hu, C.Y.J. (2026) Available online: https://BioRender.com/qv6frmb, accessed on 29 May 2026.

Overall, the central challenge for GelMA-based systems is no longer whether they can promote vascularization and osteogenesis, but whether they can achieve stable, predictable, scalable, and regulation-compatible long-term functional reconstruction in complex bone defects. Only when biological efficacy, engineering manufacturability, and clinical feasibility are met together can the potential of GelMA in vascularized bone regeneration be translated into practical therapeutic solutions. The current GelMA-based research landscape, evidence structure, key translational bottlenecks, and future roadmap for vascularized bone regeneration are summarized in Figure 6, highlighting the need to move from intrinsic programming, exogenous delivery, and stimuli-responsive design toward standardized evaluation, pathological/load-bearing models, GMP-compatible manufacturing, large-animal validation, and clinically translatable vascularized bone regeneration systems.

### 7.2. Future Prospects

#### 7.2.1. Large-Animal and Load-Bearing Validation

Future studies should move beyond rat cranial defects toward large-animal load-bearing models such as sheep, pigs, dogs, or goats. Although current GelMA studies have expanded to femoral condyle defects, pathological models, and rabbit cranial defects, true large-scale, load-bearing, and long-term validation remains lacking. Large-animal models would allow simultaneous evaluation of vascular perfusion, interface integration, bone remodeling, surgical operability, and long-term mechanical stability under cyclic loading. For load-bearing applications, GelMA may be more suitable as a vascular–osteogenic regulatory phase integrated with PCL, β-TCP, nHA, ceramic, or metallic supporting structures, rather than as an independent load-bearing component.

#### 7.2.2. Long-Term Biosafety and Degradation Matching

Long-term biosafety evaluation should extend beyond short-term cytotoxicity and major-organ histology to include chronic inflammation, immunogenicity, degradation-product metabolism, and fibrotic responses. For systems containing EVs, PRP, decellularized matrices, or nanomaterials, source heterogeneity, batch variability, and in vivo clearance pathways should also be clarified. In parallel, degradation behavior should be matched to vascular ingrowth, new bone deposition, and remodeling. Excessively rapid degradation may compromise structural support, whereas overly slow degradation may delay tissue replacement or induce persistent host responses.

#### 7.2.3. Standardized Dosing and Release Kinetics

Exogenous delivery systems require clearer standards for effective dose, safety window, release kinetics, local retention time, and batch stability for VEGF, BMP-2, DMOG, ions, EVs, and PRP/CGF. Sequential and stimuli-responsive systems should further demonstrate that release behaviors remain predictable across different batches, defect environments, and sterilization or storage conditions.

#### 7.2.4. GMP-Compatible Manufacturing and Regulatory Translation

Future GelMA systems should consider GMP manufacturing, sterilization, storage, and regulatory pathways at early stages of development. Three-dimensional printing systems should validate printing fidelity, structural stability, and batch-to-batch reproducibility after scale-up. Systems containing living cells, EVs, PRP, or decellularized matrices require standardized source tracing, quality control, and storage protocols. In addition, sterilization procedures should be systematically evaluated for their effects on GelMA crosslinking, mechanical properties, degradation behavior, and biological activity.

#### 7.2.5. Translational Roadmap

Future GelMA research should shift from “functional augmentation” toward “translational validation.” Short-term efforts should establish standardized evaluation systems for vascularization, osteogenesis, degradation, release, and mechanics; mid-term studies should prioritize pathological or load-bearing defect models; and long-term translation will require large-animal validation together with GMP manufacturing, sterilization, storage, and regulatory implementation. Among different strategies, exogenous delivery systems should prioritize dose and release standardization, and intrinsic material programming should strengthen manufacturability and mechanical adaptation, whereas stimuli-responsive systems should focus on reproducibility and clinical operability. Ultimately, successful translation will require simultaneous optimization of biological efficacy, engineering manufacturability, and clinical feasibility.

## Figures and Tables

**Table 3:** Intrinsic material programming strategies for GelMA-based hydrogels.

Strategy Dimension	Design Focus	Main Vascular–Osteogenic Role	Key Limitation	References
Spatial architecture	Pore size, pore throat size, interconnectivity, and porosity	Endothelial ingrowth and perfusable osteogenic integration	Limited temporal regulation	[52,53,59]
Matrix mechanics and degradation	Crosslinking density, stiffness, and degradation rate	Vascular reconstruction and adaptive osteogenic remodeling	Infiltration–mechanics trade-off	[57]
Spatial matrix patterning	GelMA concentration gradients or patterned compartments	Spatiotemporal vascular–osteogenic coordination	Fabrication complexity	[60]
Mechanical properties	Elastic modulus and network density	Mechanotransduction-driven vascularized bone formation	Porosity–stiffness trade-off	[61,62]
Dynamic mechanical properties	Reversible bonds, viscoelasticity, and stress relaxation	Dynamic vascular morphogenesis and mineralization	Relaxation–stability balance	[65]
Microstructural guidance	Nanotopography and aligned fibrous interfaces	Directed endothelialization and perfusion stability	Limited deep-tissue integration	[67]
Perfusable channel architecture	Channel size, connectivity, branching, and perfusion ports	Early perfusion and deep-tissue vascularization	Uncertain long-term perfusion	[69]
Interfacial chemistry	Charge, hydration, affinity motifs, and ligand density	Immuno-interfacial vascular maturation	Limited interfacial predictability	[75]
Matrix-bound bioactive presentation	Photo-crosslinkable vascular or osteogenic peptides	Sustained localized vascular–osteogenic signaling	Limited temporal adaptability	[76]

## Data Availability

No new data were created or analyzed in this study. Data sharing is not applicable to this article.

## Supplementary Materials

The following supporting information can be downloaded at: https://www.mdpi.com/article/10.3390/jfb17060281/s1, Table S1. Detailed biological regulatory cues and design implications in angiogenic–osteogenic coupling for GelMA systems. Table S2. Detailed evidence characteristics, representative models, and translational limitations of GelMA-based strategies for vascularized bone regeneration. Table S3. Detailed mechanistic effects of intrinsic material programming strategies on angiogenic–osteogenic coupling in GelMA-based systems. References [117,118,119] are cited in the Supplementary Materials.
