# Supplementary material for: Advances in GelMA Hydrogel-Enabled Angiogenic–Osteogenic Coupling: From Structural Programming to Exogenous Cue Synergy
Source: J Funct Biomater. 2026 Jun 6;17(6):281. doi: 10.3390/jfb17060281 (PMC13302648; doi:10.3390/jfb17060281)
Supplement: Supplementary file 1 [file jfb-17-00281-s001.zip › jfb-4217882-supplementary.pdf]

Table S1. Detailed biological regulatory cues and design implications in angiogenic–osteogenic coupling for GelMA systems

Key regulatory axis	Mechanism	Vascular phenotype	Osteogenic phenotype	GelMA design strategy	References
Vascular subtype specification and osteogenic niche remodeling	Type H vessels (CD31^hi/Emcn^hi) spatially co-localize with osteoprogenitors/osteoblasts	A functional microvascular niche is established and the osteogenic microenvironment is optimized	Osteoprogenitor recruitment and retention are enhanced, sustaining osteogenic activity and bone-forming capacity	Design vascular–osteogenic functional units and guide local homeostasis via material cues; prioritize functional integration rather than simply increasing vessel density	[4,5]
Temporal coupling between vascular invasion and initiation of the ossification center	During fracture repair, vascular invasion is temporally linked to establishment of the primary ossification center	Early vascular entry and persistence are prerequisites for triggering the ossification program	Impaired vascular ingrowth delays or compromises primary ossification center formation, leading to stalled repair	Early-stage scaffold architectures should be designed for invasiveness, interconnectivity, and perfusability to minimize barriers to vascular ingrowth	[27,28]
Osteoprogenitor-to-vessel feedback on morphogenesis	Osteoprogenitors migrate with vessels and shape vascular patterning via paracrine/ECM cues	Osteogenic signaling defects distort vascular architecture and weaken spatial coupling	Disrupted coupling alters invasion patterns and impairs remodeling/ossification	Preserve space for cell migration, self-organization, and matrix remodeling; avoid over-crosslinking or excessive stiffness	[6–8]
Hypoxia–HIF-1α/VEGF axis	Hypoxia stabilizes HIF-1α to activate VEGF; HIF-1α loss in osteolineage impairs angiogenesis and healing, whereas HIF activation enhances vascularized repair	Neovessel formation and network expansion are promoted, enabling perfusion for regeneration	Increased vascularization with improved bone quantity and mechanics	Prioritize endogenous repair: reduce early diffusion/ingrowth barriers and ensure connectivity/perfusion; use exogenous cues as adjuncts	[29,30]

Blood flow/perfusion as an upstream determinant	Flow modulation (ligation/pharmacology) dictates perfusion, O_2/nutrient delivery, and waste clearance	Hypoperfusion compromises vascular maturation and stability	Reduced flow decreases Osx^+ progenitors and bone formation rate	Shift from "more vessels" to perfusability and functional maintenance; prioritize channel connectivity and perfusion accessibility	[9,10]
Endothelial Dll4/Notch signaling in angiogenic–osteogenic coupling	Endothelial Dll4/Notch–RBP-J integrates cues to control phenotype and angiocrine programs	Notch sustains vascular maturation/stability and prevents non-productive angiogenesis	Notch-dependent angiocrine signals couple to osteogenic differentiation and bone formation	Engineer a niche that supports long-term endothelial homeostasis to preserve Notch–angiocrine function, not just vessel sprouting	[9–11]
Bidirectional roles of the VEGF–VEGFR axis	VEGFR1 is linked to osteogenic migration/differentiation; direct VEGFR2 functions in osteoblasts remain debated	VEGF–VEGFR2 drives angiogenesis and functional vessel formation	VEGFR blockade suppresses both angiogenesis and osteogenesis; dual blockade is additive, supporting coupled regulation	Coordinate vascular stability with osteogenesis; avoid over-amplifying a single pathway	[12–15]
Aging-driven uncoupling of angio- and osteogenesis	Aging reduces bone formation and causes bone loss, with fewer type H vessels, reduced endothelial Notch targets, and diminished perfusion; reactivating endothelial Notch restores type H vessels and improves osteogenic activity/bone mass	Decreased vascular density and function with impaired maturation/stability	Reduced osteoprogenitor activity and bone mass, limiting repair capacity	In aged niches, balance perfusion accessibility and immunocompatibility; add exogenous support when endogenous repair is insufficient	[117–119]
Inhibitory shift in the aged endothelial secretome	Aged vascular niches enrich exosomal miR-31-5p that suppresses BMSC Wnt/β-catenin; miR-31-5p inhibition rescues osteogenesis	Loss of a supportive endothelial niche	Suppressed BMSC osteogenesis and reduced bone formation	Engineer anti-inflammatory/antioxidant interfaces to avoid amplifying SASP; add exogenous antagonists when needed	[16,17]

Immune control of vascular maturation and osteogenic coupling	Acute M1 polarization initiates angiogenesis/recruitment, but sustained inflammation drives imbalance, macrophage senescence, and failed M1→M2 transition, disrupting the inflammation resolution; angiogenesis; remodeling sequence	Prolonged inflammatory/sprouting state with high risk of failed maturation and stable perfusion	Delayed repair and stalled osteogenic remodeling	Minimize foreign-body response and chronic inflammation; enable immune infiltration and phenotype switching. Avoid excessive stiffness/density and irritative interfaces that lock tissues in pro-inflammatory state	[31,32]

Table S2. Detailed evidence characteristics, representative models, and translational

limitations of GelMA-based strategies for vascularized bone regeneration

Strategy category	Key design & mechanism	Evidence level / model	Major angiogenic–osteogenic findings	Translational considerations	Ref.
Intrinsic structural programming	Bilayer GelMA-BP@Mg/GelMA-PEG-βTCP hydrogel for neurovascularized bone regeneration	In vitro/rat non-load-bearing calvarial defect	Enhanced HUVEC angiogenesis, neural differentiation, and osteogenesis with improved BV/TV and OCN expression	Multifunctional design improved neurovascular coupling but remained limited to non-load-bearing cranial models	[33]
ECM-mimicking dynamic hydrogel	DNA-network GelMA hydrogel with aptamer-mediated VEGF retention and BMSC recruitment	Rat calvarial defect	Sustained vascularization and FAK/PI3K/Akt-mediated osteogenesis	Dynamic ECM design was biologically sophisticated but manufacturing complexity and nucleic acid stability remain concerns	[37]
Prevascular structural bioprinting	PCL-nTCP scaffold containing CGF@GelMA endothelial vascular channels	In vitro + rat skull defect	Embedded endothelial channels promoted vascular ingrowth and bone repair	Structural organization closely resembled translational tissue architecture, although evidence remained limited to non-load-bearing defects	[35]
Intrinsic structural programming	Alg/BC/GelMA scaffold with aligned nanopatterns and hollow channel networks	Subcutaneous vascularization/rat calvarial defect	Hollow channels promoted CD31-positive vessel formation and bone regeneration	Cell-/factor-free design improved reproducibility and manufacturability but lacked large-animal validation	[36]
Osteon-inspired spatial programming	Dual-ring GelMA scaffold spatially organizing HUVECs and BMSCs	In vitro/large-defect model	Spatial cellular organization enhanced angiogenesis and osteogenesis	Biomimetic osteon-like architecture was promising but cell loading increased storage and regulatory complexity	[34]
Biofunctional structural bioprinting	PRP-GelMA/AlgMA/Laponite hydrogel printed within PCL framework	In vitro/rat femoral condyle defect	Promoted vascular inward growth and femoral bone regeneration	More clinically relevant geometry was achieved, but PRP variability and multi-material printing complicated	[45]

				standardization	
Exogenous cue delivery	QK peptide-functionalized GelMA/Sr-LDH@PDA hydrogel; QK mimics VEGF-related signaling and Sr-LDH supports osteogenic/vascular microenvironment	In vitro/osteoporotic bone defect model	Enhanced endothelial tube formation/migration; upregulated vascularization-related markers; promoted PDGF-BB-related Type-H vessel-associated microenvironment and BMSC osteogenesis	Pathological model strengthens relevance, but long-term safety of Sr-LDH@PDA and large-animal/load-bearing validation remain not reported	[38]
Exogenous cue delivery	AZnBg incorporated into GelMA/HA-CHO dynamic hydrogel via Schiff-base bonding; Zn-related bioactive ion release	In vitro/rat distal femoral defect	Reported HUVEC migration/tube formation and in vivo neovascularization; promoted ALP, mineralization, osteogenic gene expression and femoral bone repair	Femoral defect improves translational relevance, but large-animal evidence and long-term ion-release safety remain not reported	[39]
Exogenous cue delivery	Porous PEO-GelMA hydrogel loaded with DBM and PLGA/VEGF microspheres to combine mass transport, osteogenic cue and angiogenic cue	In vitro /ectopic VTEB/rabbit skull defect, 12 weeks	Porous structure and sustained VEGF release supported vascularization; DBM/BMSC-containing constructs enhanced OCN/CD31-positive vascularized bone regeneration and rabbit skull repair	Rabbit model and Young's modulus assessment improve evidence strength, but the system contains DBM, VEGF microspheres and optional BMSCs, increasing standardization complexity	[41]
Exogenous cue delivery	vECM/GelMA hydrogel loaded with BMP-2; vECM acts both as angiogenic component and BMP-2 affinity carrier	In vitro/rat calvarial critical-size defect, 4/8 weeks	vECM enhanced HUVEC tube formation and CD31 expression; BMP-2 delivery increased ALP/ARS/Runx2/Col I and improved BV/TV/BMD with CD31/Osx staining in vivo	Strong dual-cue logic, but vECM source variability, immunogenicity and batch consistency remain translational concerns	[42]
Exogenous cue delivery	Li-modified mesoporous	In vitro high-glucose	Li-related osteoimmunomodulatio	Diabetic model is valuable, but	[43]

	bioglass incorporated into GelMA to modulate high-glucose inflammatory microenvironment	microenvironment/diabetic rat bone defect	n promoted M2-associated VEGF/BMP-2 secretion and supported osteogenesis/neovascularization under diabetic conditions	angiogenesis is partly indirect through macrophage modulation; long-term Li/bioglass degradation safety remains not reported	
Exogenous cue delivery	3D-printable GelMA-PEGDA/SiPAC hydrogel scaffold with biodegradable SiP nanosheets releasing Si/P cues	In vitro/rat calvarial defect	HUVEC tube formation and VEGF/bFGF/CD31 expression were enhanced; in vivo CD31/VEGF staining and OCN/BMP-2 expression supported vascularized bone regeneration	3D printability and biodegradable ion cues are advantages, but it remains a small-animal non-load-bearing model; long-term nanomaterial metabolism and large-animal evidence are not reported	[40]
Exogenous cue delivery	GelMA scaffold incorporating DMOG and pearl powder; combines pro-angiogenic hypoxia-related cue with mineral/natural bioactive component	In vitro/rat femoral condyle defect, 1–3 months	DMOG/PP scaffolds improved HUVEC tube formation; in vivo CD31/α-SMA and HIF-1α increased alongside OPN/OCN, micro-CT and histological bone repair	Femoral condyle and 3-month follow-up strengthen evidence; however, natural PP batch variability, DMOG dose control and long-term systemic safety require further validation	[44]
Temporally programmed delivery	GelMA-VEGF combined with vascular ECM-PCSK9 to separate early pro-angiogenic stimulation from later osteogenic regulation	In vitro/in vivo bone defect model	VEGF supported early vascularization, while PCSK9 promoted BMMSC osteogenesis through ERK-related signaling	Mechanistically novel but evidence for load-bearing defects, large animals, and long-term safety was not reported	[46]
Spatiotemporally graded organoid system	GelMA matrix carrying BMSCs/HUVECs/DMOG with SilMA/nHAp microspheres; rapid GelMA degradation initiates prevascularization,	In vitro dynamic organoid culture/ectopic implantation/mouse critical-sized calvarial defect	Sequential coupling improved vascular perfusion and bone matrix deposition; Osterix and VEGF-A increased, with critical-sized calvarial repair	Strong time-sequence logic and organoid relevance, but clinical translation is limited by cellular complexity, scale-up, GMP manufacturing, and non-load-bearing model	[49]

	while sustained microsphere degradation supports osteogenic maturation				
Progressive factor release within structural scaffold	3D-printed PCL/nHA/Laponite scaffold filled with covalently tethered GelMA-VEGF hydrogel to combine mechanical support and controlled VEGF release	In vitro/rat critical-sized calvarial defect	Sustained VEGF availability promoted endothelial survival/vascularization, while PHL framework supported osteogenic differentiation and bone repair	More engineering-oriented than simple delivery systems, but real load-bearing validation, large-animal evidence, and long-term release safety were not reported	[47]
Spatiotemporally responsive / piezoelectric scaffold	Dual-nozzle 3D-printed BTO/nHA/PCL scaffold combined with metformin-loaded GelMA hydrogel; piezoelectric response and drug release coordinate early angiogenesis and later osteogenesis	In vitro co-culture/dual animal bone defect models	Early endothelial migration/tubular network formation and extensive neovascularization; MSC osteogenesis at 14/21 d and accelerated osseous tissue infiltration	Highly aligned with sequential coupling, but depends on piezoelectric materials, ultrasound stimulation, drug-release parameters, and complex manufacturing consistency	[50]
Sequential exogenous cue release	HAMA/GelMA Janus microcarriers compartmentalizing VEGF and BMP2; HAMA hemisphere rapidly releases VEGF, while GelMA hemisphere sustains BMP2 release, with magnetic localization	In vitro/rat cranial defect model	Rapid VEGF release promoted early vascular network formation; sustained BMP2 release supported later osteogenic differentiation, with increased CD31, OPN and OCN in vivo	Clear temporal design, but microcarrier localization, release-dose control, magnetic enrichment, and scalable manufacturing remain translational barriers	[48]

Table S3. Detailed mechanistic effects of intrinsic material programming strategies on angiogenic–osteogenic coupling in GelMA-based systems

Strategy dimension	Material design	Mechanistic chain	Vascular effects	Osteogenic effects	References
Spatial architecture	Pore size distribution, pore throat dimension, interconnectivity, porosity	Reduced migration and transport barriers; deeper endothelial infiltration and 3D network formation; establishment of perfusable networks; improved maturation and stability	Greater ingrowth depth; higher network continuity; enhanced maturation and stability	Improved mass transport; more stable onset of mineralization; better tissue integration	[52,53,59]
Spatial architecture	Crosslinking density, stiffness, and degradation rate	Light crosslinking/soft matrices preserve open porosity and support infiltration/integration; bias macrophages toward a pro-repair phenotype and dampen inflammation; favor integration over encapsulation, reducing fibrosis risk	Enhanced vascular ingrowth and network reconstitution; higher success of stable perfusion; lower risk of impaired maturation/perfusion maintenance	Smoother repair progression; timely osteogenic onset and sustained bone formation; reduced risk of delayed osteogenesis	[57]
Spatial architecture	Concentration tuning; spatial partitioning and patterning	Spatially patterned GelMA with distinct concentrations to separate early permissive ingrowth from long-term structural support, minimizing functional conflicts and reducing uncoupling risk	Faster early vascular ingrowth; improved network organization and maturity	Stabilized mineralized core; improved continuity of integration; reduced risk of insufficient early invasion or inadequate late-stage support	[60]
Mechanical properties	Elastic modulus/network density	Too soft: insufficient support and pore collapse, unstable sprouting and fragmented networks; too stiff: restricted cell rearrangement, impaired extension and anastomosis; an optimal modulus balances support with remodelability	Improved connectivity and anastomosis; higher success of mature vessels and stable perfusion	Mechanotransduction (e.g., YAP/TAZ) regulates osteogenesis; in GelMA, moderately increased modulus with optimized porosity enhances osteogenic markers and in vivo bone formation	[61,62],[64]

Mechanical properties	Viscoelasticity and stress relaxation	Introduces reversible bonds to enable stress relaxation; uses a secondary network for shape retention; moderates relaxation and promotes integrin activation, endothelial spreading, and network formation, whereas excessive relaxation suppresses sprouting	Enhanced endothelial spreading, sprouting, and network assembly; at comparable stiffness, faster relaxation more effectively triggers vascular morphogenesis	ECM-like dynamic mechanics promote osteogenic differentiation and mineralization; covalent-only GelMA relaxes poorly, and dynamic networks compensate effectively	[65]–[66]
Organized microstructures	Nanotopography/aligned fibrous interface	Fibrous nanotopography enhances cell spreading and directional migration, tightens junction organization, and improves endothelial monolayer integrity and endothelialization	More complete and continuous endothelialization; easier formation of an intact endothelial monolayer	Structural basis for stable perfusion and improved tissue integration	[67]
Organized microstructures	Channel architecture design	Tunes channel diameter, spacing, connectivity, and branching topology; integrates perfusion ports; supports luminal endothelialization. Shortened diffusion distance and convective transport relieve deep hypoxia, accelerate endothelialization, and stabilize lumens, enabling a "perfusion-first, microvessel-later" build-up	Faster perfusion establishment; improved lumen stability; better interconnection in thick/deep regions	Improved deep-cell survival and osteogenic niche; more continuous mineralization, reducing repair interruption	[69],[71],[72]

Chemical interface	Charge/hydrophilicity/affinity sites/ligand density	Tuning interfacial charge, hydration, affinity motifs, and ligand density reshapes early protein adsorption and enriches specific binding proteins; dampens inflammatory signaling (e.g., NF-κB), reduces immune activation, and promotes endothelial migration, anastomosis, and maturation, improving controllable vascularization while supporting osteogenesis	Network formation with a maturation-prone phenotype; improved stability and controllability, avoiding "sprouting-only" angiogenesis	Improved osteoblast adhesion, expansion, and differentiation niche; reduced chronic/pro-inflammatory cues, relieving osteogenic inhibition and supporting smoother mineralization and integration	[75],[79]
Chemical interface	Co-crosslinking of bioactive ligands and matrix-bound presentation	Co-crosslinks photo-crosslinkable peptides with GelMA to anchor cues within the network, minimizing burst loss/inactivation; emphasizes matrix-bound presentation rather than free diffusion	GelMA supports vascularization, but soluble VEGF provides transient stimulation; co-crosslinked VEGF-mimetic peptides markedly enhance endothelial responses and capillary-like network formation	GelMA–c–OGP enables sustained osteogenic cue presentation, upregulating BMP-2, OCN, and OPN, increasing mineral deposition, and improving in vivo bone formation/repair	[76],[77]
